# Supplementary material for: Causal Relationship between Aging and Anorexia Nervosa: A White-Matter-Microstructure-Mediated Mendelian Randomization Analysis
Source: Biomedicines. 2024 Aug 16;12(8):1874. doi: 10.3390/biomedicines12081874 (PMC11351342; doi:10.3390/biomedicines12081874)
Supplement: Supplementary file 1 [file biomedicines-12-01874-s001.zip › Supplementary Figure/Figure S4/Singe SNP plot of MR analysis for TL to ubm-b-1528.pdf]

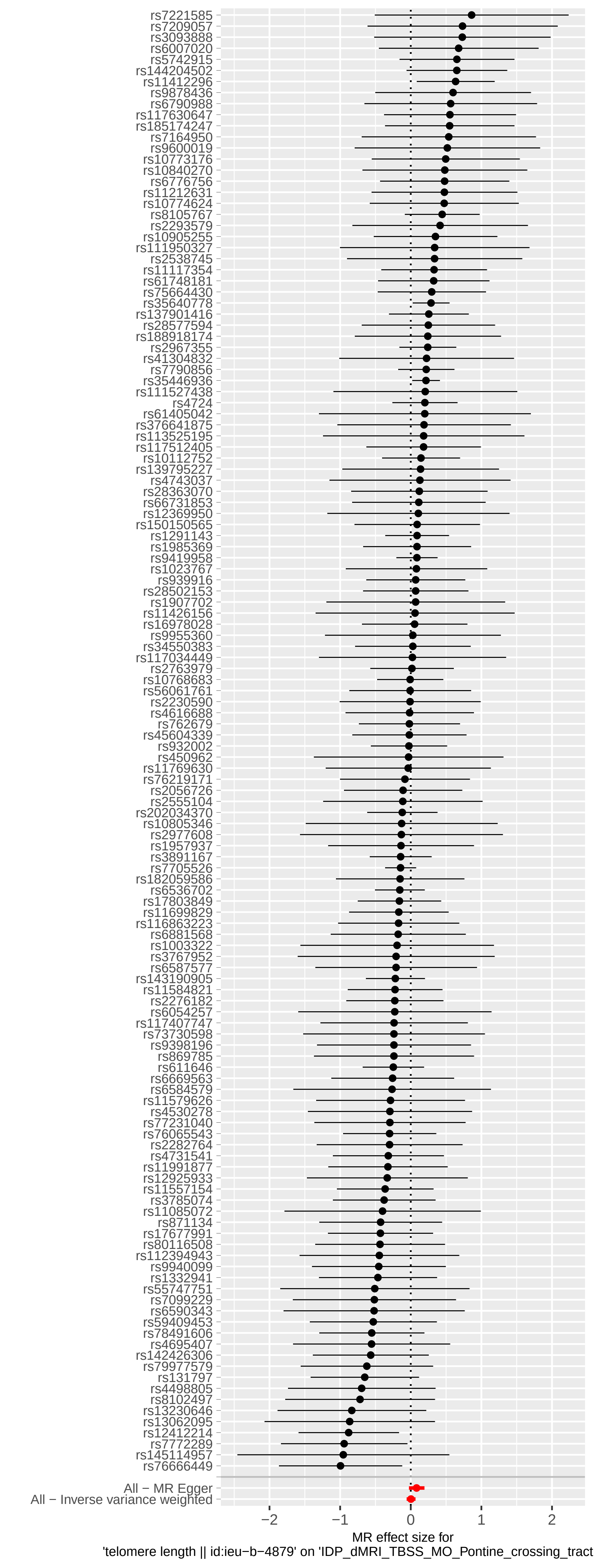
rs7221585
rs7209057
rs3093888
rs6007020
rs5742915
rs144204502
rs11412296
rs9878436
rs6790988
rs117630647
rs185174247
rs7164950
rs9600019
rs10773176
rs10840270
rs6776756
rs11212631
rs10774624
rs8105767
rs2293579
rs10905255
rs111950327
rs2538745
rs11117354
rs61748181
rs75664430
rs35640778
rs137901416
rs28577594
rs188918174
rs2967355
rs41304832
rs7790856
rs35446936
rs111527438
rs4724
rs61405042
rs376641875
rs113525195
rs117512405
rs10112752
rs139795227
rs4743037
rs28363070
rs66731853
rs12369950
rs150150565
rs1291143
rs1985369
rs9419958
rs1023767
rs939916
rs28502153
rs1907702
rs11426156
rs16978028
rs9955360
rs34550383
rs117034449
rs2763979
rs10768683
rs56061761
rs2230590
rs4616688
rs762679
rs45604339
rs932002
rs450962
rs11769630
rs76219171
rs2056726
rs2555104
rs202034370
rs10805346
rs2977608
rs1957937
rs3891167
rs7705526
rs182059586
rs6536702
rs17803849
rs11699829
rs116863223
rs6881568
rs1003322
rs3767952
rs6587577
rs143190905
rs11584821
rs2276182
rs6054257
rs117407747
rs73730598
rs9398196
rs869785
rs611646
rs6669563
rs6584579
rs11579626
rs4530278
rs77231040
rs76065543
rs2282764
rs4731541
rs11991877
rs12925933
rs11557154
rs3785074
rs11085072
rs871134
rs17677991
rs80116508
rs112394943
rs9940099
rs1332941
rs55747751
rs7099229
rs6590343
rs59409453
rs78491606
rs4695407
rs142426306
rs79977579
rs131797
rs4498805
rs8102497
rs13230646
rs13062095
rs12412214
rs7772289
rs145114957
rs76666449
All − MR Egger
All − Inverse variance weighted
−2
−1
0
1
2
MR effect size for
'telomere length || id:ieu−b−4879' on 'IDP_dMRI_TBSS_MO_Pontine_crossing_tract
